# Towards a digital twin for supporting multi-agency incident management in a smart city

**DOI:** 10.1038/s41598-022-20178-8

**Published:** 2022-09-28

**Authors:** Kristina Wolf, Richard J. Dawson, Jon P. Mills, Phil Blythe, Jeremy Morley

**Affiliations:** 1 School of Engineering, Newcastle University, Newcastle upon Tyne, NE1 7RU UK; 2 Tyndall Centre for Climate Change Research, Newcastle upon Tyne, UK; 3 Ordnance Survey, Southampton, SO16 0AS UK

**Keywords:** Natural hazards, Environmental impact, Environmental health, Sustainability

## Abstract

Cost-effective on-demand computing resources can help to process the increasing number of large, diverse datasets generated from smart internet-enabled technology, such as sensors, CCTV cameras, and mobile devices, with high temporal resolution. Category 1 emergency services (Ambulance, Fire and Rescue, and Police) can benefit from access to (near) real-time traffic- and weather data to coordinate multiple services, such as reassessing a route on the transport network affected by flooding or road incidents. However, there is a tendency not to utilise available smart city data sources, due to the heterogeneous data landscape, lack of real-time information, and communication inefficiencies. Using a systems engineering approach, we identify the current challenges faced by stakeholders involved in incident response and formulate future requirements for an improved system. Based on these initial findings, we develop a use case using Microsoft Azure cloud computing technology for analytical functionalities that can better support stakeholders in their response to an incident. Our prototype allows stakeholders to view available resources, send automatic updates and integrate location-based real-time weather and traffic data. We anticipate our study will provide a foundation for the future design of a data ontology for multi-agency incident response in smart cities of the future.

## Introduction

Digital Twins are digital representations of the real-world, including physical objects, systems, and processes, that aid in modelling and monitoring entire cities, their relationships and behaviour^1^. Liu et al. provide a comprehensive literature review on different “digital twin” terminology found nowadays in various sectors^2^. Examples of digital twins include asset digital twins of machines to understand their condition^3^; digital twins of components such as sensors to test different real-world behaviours in a simulated environment^4^; system digital twins to monitor the behaviour of systems network such as power plants^5^; or process digital twins of business processes that simulate process flows^6^, including the movement of people or goods. Digital twins can have different maturity levels depending on which functionalities they support^7,8^:

- Supporting the full product life cycle management from design to production;
- Enabling real-time monitoring and control;
- Optimising operational workflows; and providing predictive and preventive maintenance.

The recent development and rise in internet-enabled devices, Internet-of-Things, and cost-effective solutions designed to store and manage frequent incoming big data streams ease the collection and analysis of this (near) real-time information^9^. Similar to Kamilaris et al. we refer to Things as interconnected physical devices that collect high-resolution data of physical city entities, monitor changing conditions, and support early warning mechanisms^10^. In line with the United Nations Sustainable Development Goal (SDG) 11, to make cities inclusive, safe, resilient, and sustainable, smart cities require the ability to better respond to and be prepared for unforeseen events^11^. We refer to resilience as “reducing the impacts resulting from disturbance”^12^, i.e. the ability of a system to resist or to tolerate disturbance, to adapt and respond to changing conditions, to recover from challenges (crisis or disasters), and to move forward quickly^13^. To reduce the overall disturbance and support resilience in smart cities, we can use real-time data to monitor the environment, detect early incidents and distribute warnings across the many interconnected systems and governance boundaries where incidents might occur or impact, such as in smart cities^14^.

We define a smart city as a “system of systems” consisting of multiple, heterogeneous, distributed components that can interact and exchange information across a large-scale network^15–17^. Smart cities use information and communication technologies, remote sensing and insitu sensors to autonomously collect and store current information on environmental conditions^18–21^. As the knowledge of a digital twin grows over its lifecycle, it can help generate significant long-term value from raw data across different domains^22^, such as transport: Traffic signals connect to vehicles equipped with on-board units to adjust the green signal, thus, improving bus service for passengers and reducing congestion at key intersections^23^; Environment: Sensors measure the air to implement measures to curb pollution and improve quality of life^24^; Public safety: Personal hazard alerts triggered by proximity systems help to inform construction workers of potential risks, such as overhead dangers, i.e. creating a safer working environment^25^; Infrastructure: Sensors help to monitor the condition of infrastructures, e.g. sewers in flood-prone areas, to provide timely notification of potential flooding events^26^. Currently, such smart city applications are usually independent systems that run isolated and do not exchange data across their systems with other domains^22^. For a digital twin to realise its full benefit, it must enable information sharing and collaboration vertically within the company’s information systems and horizontally across multiple stakeholder groups^22^. Connecting interdependent data across these boundaries can provide a more comprehensive operational picture and enable more efficient and informed decision support, which demonstrates the real opportunity of what a smart city can deliver.

Further challenges relate to data requirements, as incidents can become quite complex and the demands for the necessary data increase. In the first instance, stakeholders involved in incident response require static data existing prior to the incident, such as topography, buildings, administrative borders, census data including vulnerable population groups, existing hazard maps, and critical infrastructures such as gas, water, and electricity^27^. Moreover, as many incidents are dynamic in nature, i.e., change with time, further dynamic and temporal data is needed to provide a more comprehensive operational picture, such as incident type, scale and impact area, people involved, potential casualties, injuries and fatalities^27^. To capture the rapidly changing condition of an incident and support time-critical response, we can extract data streams, e.g. from stationary weather stations, water gauges, traffic sensors, and mobile phones^18,20^. Such internet-enabled devices record a series of spatial events showing the physical location through geographic coordinates (e.g. longitude and latitude), attributes describing the observation recorded, and a timestamp. Due to heterogeneous sources, incoming data may be in a structured format showing figures of impacted buildings, causalities, injuries, demographics, location-based coordinates, and sensory data (temperature, humidity, wind speed, and precipitation), semi-structured or unstructured, such as multi-media data (images and videos), social-media tweets, and online news data^21^. A major smart cities challenge is in connecting these different physical devices, aggregating and analysing raw data to provide information that enables a holistic understanding of the current incident condition^28^. This fusion and management of heterogeneous data streams and the required assessment in real-time can lead to several challenges, which are classified by the literature as the three big Vs^29^: Volume of real-time data can vary depending on the available data sources; (2) Velocity of data streams can differ (e.g. data can arrive periodically or continuously); and (3) Variety can depend on the data origin, resulting in incompatible data formats.

This study contributes to research on how to integrate heterogeneous data between distributed multi-agency emergency systems into a common data model; how to apply processing steps to continuous real-time data streams; and how to analyse and visualise location-based incident data to enhance the understanding of the current incident environment. This study emphasises multi-agency collaboration, integrating different types of data from various agencies to other agencies and showing how best to achieve meaningful interpretation of the heterogeneous data through an integrated incident response workflow. What we show in this study using the multi-agency incident response example can be applied to many other multi-agency environments.

To demonstrate how to address challenges associated with digital twin technology, we propose a prototype of a process digital twin that supports real-time monitoring and provides initial operational support through data information sharing and collaboration across different organisational and system boundaries. Following the systems engineering approach, we ensure that the system is developed with the diverse multiple end-users in mind achieved through continuous stakeholder engagement. The systems engineering method has already been used for different types of engineering problems, from the beginning of the product lifecycle to the post-development phase, such as production and operational support^30^. NASA has been applying the systems engineering methodology since 1995 to improve product development and delivery for human spaceflight, robotic, aircraft, or ground-based technology projects in complex project environments with multiple stakeholders involved^31^. McGee and McGregor demonstrate how systems engineering can be used to develop Intelligent Transportation Systems that need to support data flow between systems and enable data analysis from connected vehicles at scale^32^. We further enrich this method by applying more specifics around innovative spatial methods, such as data integration, which proves a specific methodological advance.

## Methods

Based on the concept that a smart city, a system of systems, is comprised of several autonomous entities, this study adapts parts of the systems engineering methodology^33^. A detailed description of the individual steps can be found in the Full Methods section.

## Results

### Defining the research problem

Despite the advances in internet-enabled technology, cloud-based computing resources and practical multi-agency incident response, multi-agencies encounter heterogeneous data sources, proprietary systems and the lack of real-time data collection^34^. However, real-time data such as traffic, weather, and flood information can help coordinate multiple services, such as navigating an emergency vehicle to a site where an accident has occurred somewhere en route. To better understand how a system design for a unified response system can help in the future, we first define the problem based on the literature^20,27^ and feedback discussions with stakeholders. First, we look at the generic steps involved in incident response (1-4): (1) Incoming call: The incident starts with a 999 (UK emergency number) call that is dispatched to the control centre in the corresponding area of the emergency service authority and answered by the operator. (2) Mobilisation stage: The operator records the incident type and location. Based on the incident type, the system proposes a predetermined attendance (PDA) and an estimated time of arrival (ETA). (3) Preliminary situation assessment: When the first responder vehicle arrives at the incident site, the responders manually update their status via a mobile tablet, which is visible to the control room. (4) Intervention phase: Responders execute the operational emergency response. If further support is required, they communicate with other blue light (emergency) services or other stakeholders via telephone or radio. Major incidents can quickly become highly complex and require tasks to be coordinated across a distributed network^35^. Different processes will be activated based on the incident at hand, all requiring various kinds of data. The UK introduced the Joint Emergency Services Interoperability Principles (JESIP) in 2012 to support multi-agency interoperability and improve the way the police, fire and rescue, and ambulance services collaborate^36^.

### Identifying stakeholder needs

The UK Civil Contingencies Act 2004 (Part I) constitutes roles and responsibilities involved in emergency preparation and response and classifies Category 1 (fire and ambulance services, police, local authorities, Environment Agency, and NHS bodies) and Category 2 responders (co-operating bodies responsible for their sector, e.g. utilities, telecommunications, and transport companies). Table 1 shows the status of the critical challenges in multi-agency response identified by the consulted stakeholders including North East Ambulance Service, Tyne and Wear Fire and Rescue Service, Northumbria Police, Environment Agency, North Yorkshire County Council, Local Resilience Forum Leicester, Highways England, Department for Transport, North East Urban Traffic Management and Control, Traffic Accident Data Unit (based at Gateshead Council), Government Office for Science, ResilienceDirect, Northumberland National Park Mountain Rescue Team, Ordnance Survey, and Urban Observatory Newcastle.

**Table 1:** Challenges in multi-agency response (own table, based on discussions held with stakeholders listed in subsection Identifying stakeholder needs).

Area	Challenge	Description
Processes	Redundancies in communication	Communication between key responders runs through multiple control centres, hindering efficiency in responding to incidents
	Inefficiencies in information collection	Key responders at a scene cannot see and do not know when other responders will arrive. They obtain this information from the control centre, making it difficult to create a real-time operational picture for all responders
	Missing joint overview across agencies	Key responders do not have a common view of resources allocated to the incident, making it difficult to assign incident-relevant information across agencies
People	Silo working cross agencies	Silo work occurs internally and externally, as different processes and procedures are in place. Due to competing goals, these different processes do not always seem to be compatible right away
Data	Lack of sharing information	Relevant incident data is not always shared with other agencies, making it challenging to act proactively
	Lack of real-time information	Real-time data, e.g. traffic, weather and flooding information, is not available. First responders on their way to an incident do not know if another road traffic accident has occurred on their route, which may hinder a timely response
Data	Heterogeneous data landscape	Multi agencies have various different system providers, making it difficult to ensure interoperability externally with other agencies
Technology	Lack of third-party software extensions	Existing software is often proprietary, making it challenging to link further third-party software extensions that could be useful for multi-agency response collaboration
Analysis	Lack of real-time analytics	Data analysis is mainly a historical data evaluation of past events and involves little or no real-time data or does not take predictive analysis into account

The table organises the identified stakeholder needs into the areas: Processes, People, Data, Technology, and Analytics. Each area lists the current challenges and describes them in more detail.

### Analysing functional requirements

Based on semi-structured discussions held with stakeholders involved in incident response, we developed a stakeholder analysis catalogue holding functional and technical requirements for the design of an incident response system. Table 2 shows how the different gaps identified in Table 1 can be addressed and how responders can benefit from these improvements. In further steps, this catalogue of requirements will be expanded with further discussion results from stakeholders and requirements for systems engineering according to ISO/IEC 25010 as defined by the International Organization for Standardization (ISO) and the International Electrotechnical Commission (IEC)^37^.

**Table 2:** User requirements for multi-agency incident response (own table, based on discussions held with stakeholders listed in subsection Identifying stakeholder needs).

Area for improvement	Requirement description ("As a key responder, they aim…")	Added value ("Key responders…")
**Process:** Inefficient data	To send the estimated time of arrival at the incident site to and receive from others	Know at the incident site when to expect further resource support
**Process:** Inefficient data	To view the updated incident status in the incident response system	See immediate real-time data through automated system updates
**Process:** Missing joint view	To have a cross-agency view of available and allocated resources	Have a single point of source showing the most relevant data
**Process:** Missing joint view	To monitor the first responder vehicle along the way to the incident scene	Know when to expect further resources to support at the incident site
**People:** Communication	To receive a notification when the first responder vehicle arrives at the incident site and leaves the incident site	Depend less on multi-step phone-based communication
**People:** Communication	To send and receive automatic updates via a common system	Depend less on phone due to automated system updates
**Data:** Lack of real-time information	To view integrated real-time traffic (e.g. accidents that can impact the fastest route to the incident site) and weather data	Incorporate external hazards in the decision making process that can impact the incident handling
**Technology:** Heterogeneity	To view data of different formats and sources	Access and view heterogeneous datasets
**Analytics:** Lack of real-time analytics	To have access to real-time data analytics	Incorporate historical patterns and real-time insights

The table shows the functional requirements for each area of improvement: Processes, People, Data, Technology, and Analytics. In addition, the table shows the benefits and added value of the implemented functionalities for the stakeholders involved in the incident process.

### Designing incident response system

**Figure 1:**
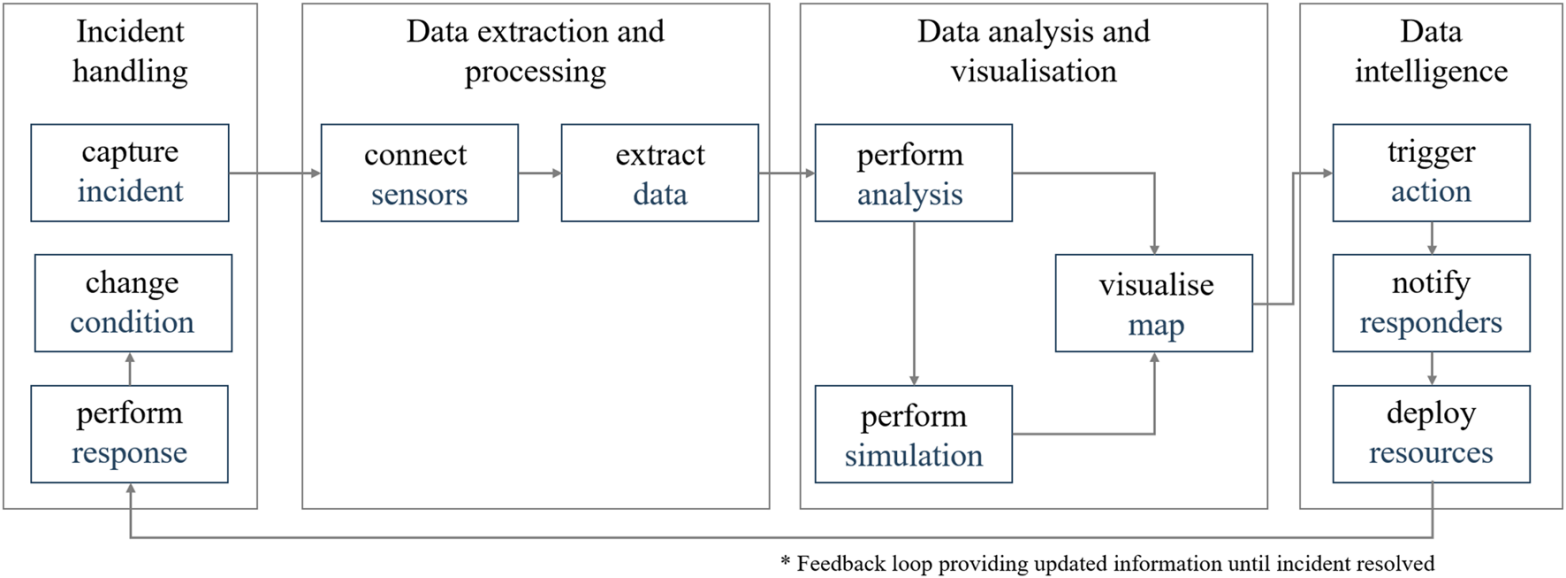
Conceptual design for the digital twin for incident response (own figure created using Microsoft PowerPoint, based on Ford and Wolf ^19^). The design of the customised model includes the stages: Incident handling, data extraction and processing, data analysis and visualisation, and data intelligence. The arrows indicate the sequential flow between different steps.

Ford and Wolf propose a conceptual model of a smart city digital twin (SCDT) for disaster management and administration that enables data sensing and simulation across diverse systems^19^. A digital twin is a digital replica of physical city assets^38^ that aids in modelling and monitoring incident response within a smart environment and improving resilience management^39^. The SCDT proposed by Ford and Wolf consists of three components^19^: (1) Components outside of the SCDT that incidents impact; (2) Smart city component: Internet-enabled devices, such as sensors generate data that users can use for analysis and simulation in the digital twin; and (3) Digital twin: Digital images and real-world infrastructure features are used to predict future conditions. Lessons learned from the SCDT can improve decision-making and actions, leading to the desired change of the current environment’s condition. In contrast to individual infrastructure systems, an emphasis of this proposed model is on iterative feedback loops. These feedback loops arise from the interdependencies and interactions of individual autonomous systems. Feedback iterations can change the state of the environment. For our study, we adapt a modified version of this concept. Figure 1 visualises the conceptual design of the adapted model for the incident response system^19^.

As shown in Fig. 1, the workflow is triggered as soon as an incident occurs. The next step is to connect to internet-enabled devices and extract raw data. We process the data, for example, by extracting the value of the measured variable, location coordinates, and the timestamp, and perform various analyses and simulations. Examples include the spatial analysis of the incident location and the route from an available responder vehicle to the incident site, including possible traffic accidents or weather hazards. The resulting outcomes are displayed visually in the form of a map. As a further step, we can support stakeholders with more intelligent data insights on which they can decide action plans, notify responders and deploy resources. After first responders have arrived at the incident site, stakeholders can monitor whether the conditions of the incident change. Until the incident is resolved, stakeholders involved in the incident response receive updated information in a feedback loop.

**Figure 2:**
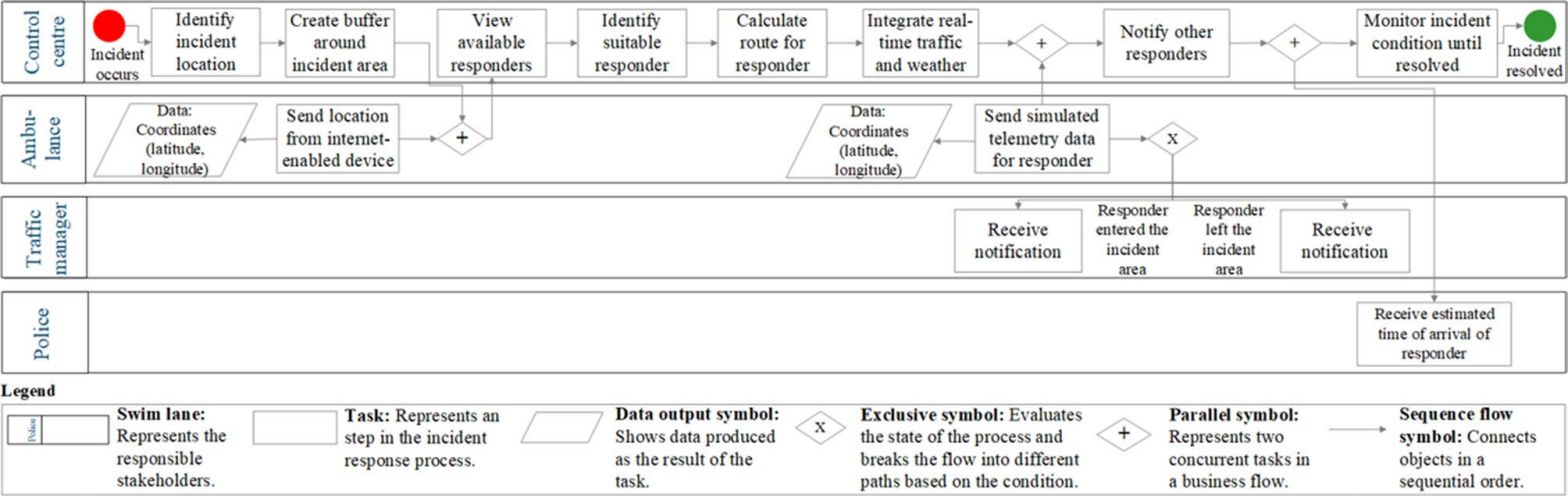
Incident process model for use case (own figure created using Microsoft Visio). The open standard of Business Process Model and Notation (BPMN) is used to depict the steps involved in the incident response use case.

To demonstrate how the adapted SCDT model^19^ can help to address current stakeholder challenges, this study develops a use case involving an accident on the Tyne Bridge, a critical location transport link over the River Tyne in North East England. One incident can impact two cities at once, Newcastle upon Tyne and Gateshead. After an incident has occurred and various processes have been initiated (depending on the incident type), stakeholders can raise different questions. The police want to know: Where is the current nearest first responder vehicle? or what is the estimated time of arrival? The traffic manager, who is responsible for monitoring traffic cameras and informing the public about road closures, wants to know: When have the responder vehicles left the site of the accident? or when can the road be reopened, and traffic resume its ordinary course? To answer such questions, and to help other responders during the incident process, responders can use the developed incident response system for the following processes: (1) Indicate the incident location; (2) Indicate the surrounding incident area; (3) Identify any impacted assets in the incident area; (4) Identify the shortest route to the incident site for the responder vehicle; (5) Simulate a responder vehicle equipped with an internet-enabled device on the way to the incident; (6) Monitor when the responder vehicle arrives at the incident using telemetry data; (7) Send a message to other stakeholder groups when the responder vehicle arrives at the incident site; (8) Monitor when the responder vehicle leaves the incident site; (9) Send a message to stakeholders when the vehicle leaves the incident site. Figure 2 shows an incident process model with different responsibilities and steps involved based on the developed use case.

### Feedback on system design

The stakeholder requirements identified so far serve as a basis for an initial prototype of the incident response system. Using a first feedback cycle, we showcased the implemented functionalities to the stakeholders in a demo presentation. The common feedback was that the functionalities are helpful and can support the challenges in the current incident response process. Further, feedback suggested to base the calculation of the closest responder not only on network distance but also on estimated time of arrival, as different roads (e.g. rural road and motorway) determine how quickly a vehicle might reach an incident site.

### Developing prototype

We implement the workflow described in Fig. 2 using the cloud computing capabilities provided by Microsoft Azure^40^, which offers a collection of geospatial services for web and mobile application under Azure Maps. We use a C# application to simulate location data for an internet-enabled device of a responder vehicle along a network route^41^. We run the application in Microsoft Visual Studio with the .NET Core SDK 3.1 on our development machine. We develop a customised web application using Azure Maps, HTML5, JavaScript, and CSS and use various Representational State Transfer (REST) Application Programming Interface (API) services to integrate live traffic, incidents, weather, and hazards^42^.

For connecting data across different platforms and combining static and dynamic data, we propose different methods. Basic data from publicly available platforms are available as .geojson, a format used for geographic data structures, e.g. points, lines and polygons, or as Web Feature Services (WFS), which contain data about geometries and attributions of individual features. We extract open data on road networks and administrative areas, e.g. local authorities that provide additional information for the study area, such as district names. Geojson objects can be loaded directly into the javascript file of the system prototype. Alternatively, we can use Azure data storage**, **such as Binary Large Objects (BLOB) storage^43^ and reference the path to the data in the javascript file. Real-time data streams are included using REST API services to provide current updates on weather, transport and hazards.

**Figure 3:**
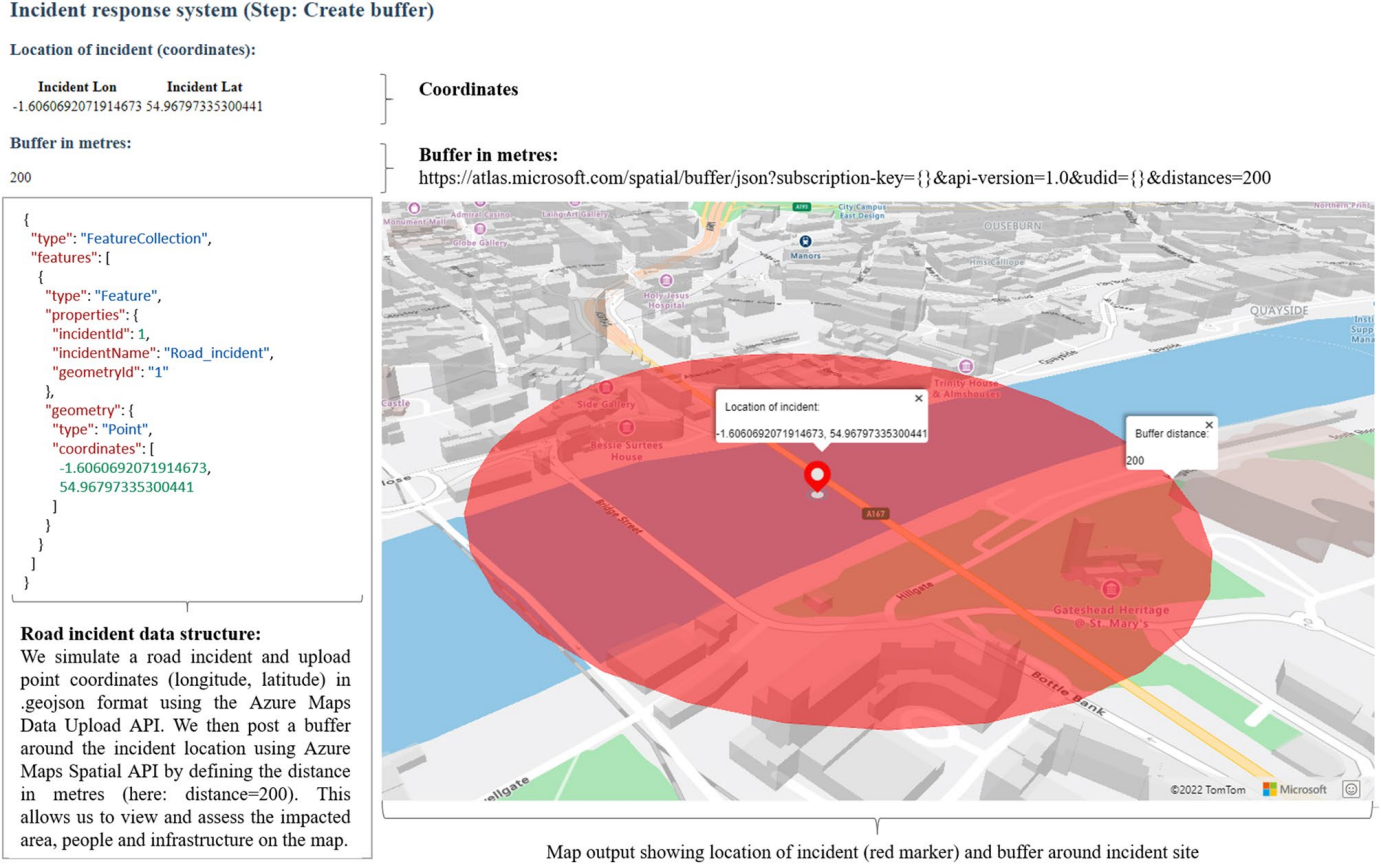
Incident process steps 1–2: Get incident location and create buffer (own figure). The web application shows the indicators: Location of incident (longitude, latitude) and buffer in metres. The code excerpt displays a .geojson file containing information for a simulated incident (incident ID, incident name, longitude, and latitude coordinates). The map output visualises the location of the incident on the Tyne Bridge with a red marker and a red circular buffer area around the incident location. KW created the map output using the Microsoft Azure Maps Web Software Development Kit and TomTom (^©^ 2022) base map data (a subscription key to use the data can be obtained after registering on the Azure Maps platform for Geospatial Mapping APIs under https://azure.microsoft.com/en-us/services/azure-maps/#azuremaps-overview). The code for the web application was developed in JavaScript, CSS and HTML in the Microsoft Visual Studio Code integrated development environment (version 1.70.2) and Node.js (version 16.13.2) on a Windows NT 64-bit operating system (×64-based processor)^40^.

The customised web application implements the following steps (1-8): (1) *Detect the incident*: We simulate a road traffic incident and upload the incident details (incident ID and incident type) and coordinates (longitude, latitude) in .geojson using the Azure Maps Data Upload API. Stakeholders can then view a point marker at the incident location on the map. (2) *Identify the incident area*: We post a buffer around the incident location using Azure Maps Spatial API by defining the distance in metres (here: distance = 200). (3) *View the available resources*: We simulate the location coordinates (longitude, latitude) and details of different responders (responder ID, responder name) in a geojson file using Azure Maps Data Upload API. This allows stakeholders to view the location of different responders on the map. (4) *Identify the closest responder*: Using Azure Maps Spatial API, we can identify the closest responder for the fastest incident response. (5) *Calculate the responder route to the incident*: With the location of the incident and the location of the closest responder, we can calculate the route to the incident using the Azure Maps Route Service API. (6) *Analyse real-time traffic and weather data*: We use the Azure Maps AccuWeather service and Azure Maps TomTom traffic service to show stakeholders real-time traffic accidents, road closures and other known hazards along the route. (7) *Simulate an IoT-enabled device*: We upload a polygonal area in .geojson using the Spatial Post Geofence API. We simulate telemetry data from a responder vehicle along a given network route using Microsoft Visual Studio, C# and the .NET framework. The coordinates help to identify if the responder vehicle is in proximity to the incident site, has entered the geofence (incident) area, or has left. (8) *Monitor when responders are within the geofence*: Using the Azure geofencing function, we can define the geographical area of the incident and determine whether a first responder vehicle has moved within the area. When the responder enters the incident site, traffic managers receive a notification (e.g. in the form of a text), telling them when the responder arrives at the incident to close the road. When the responder vehicle exits the incident site, traffic managers receive another notification, saying that the responder left, and traffic can resume. Figure 3 visualises the output of the system prototype for the steps 1 and 2 of the incident response process.

**Figure 4:**
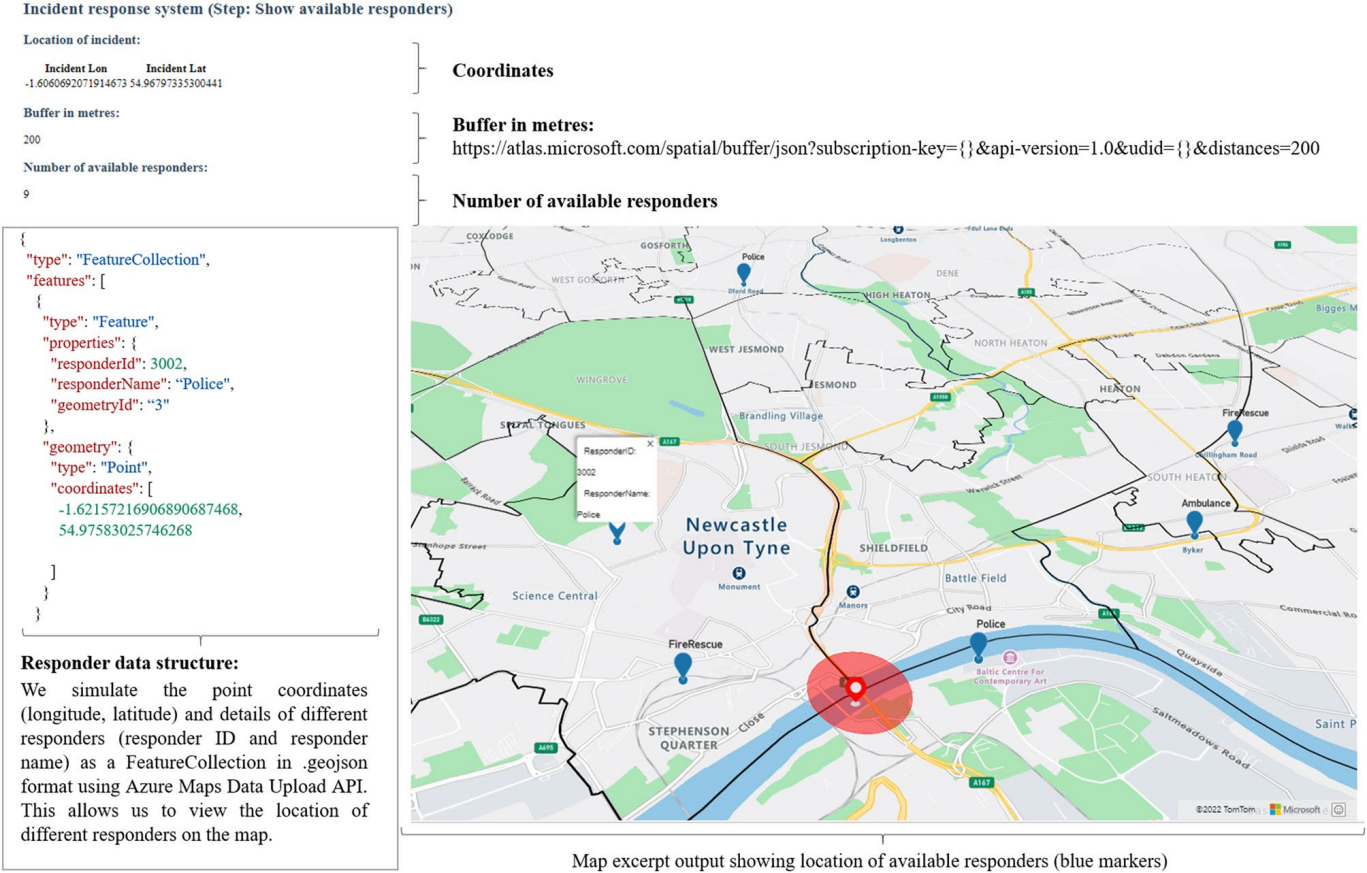
Incident process step 3: View location of available responders (own figure). The web application shows the indicators: Location of incident (longitude, latitude); buffer in metres; and the number of available responders. The code excerpt displays a .geojson file containing the information for a simulated responder (responder ID, responder name, longitude and latitude coordinates). The map output visualises the location of the incident on the Tyne Bridge with a red marker and the location of available responders with blue markers. KW created the map output using the Microsoft Azure Maps Web Software Development Kit and TomTom (^©^ 2022) base map data (a subscription key to use the data can be obtained after registering on the Azure Maps platform for Geospatial Mapping APIs under https://azure.microsoft.com/en-us/services/azure-maps/#azuremaps-overview). The code for the web application was developed in JavaScript, CSS and HTML in the Microsoft Visual Studio Code integrated development environment (version 1.70.2) and Node.js (version 16.13.2) on a Windows NT 64-bit operating system (×64-based processor)^40^.

Figure 4 shows the output of the system prototype for step 3 of the incident response process.

**Figure 5:**
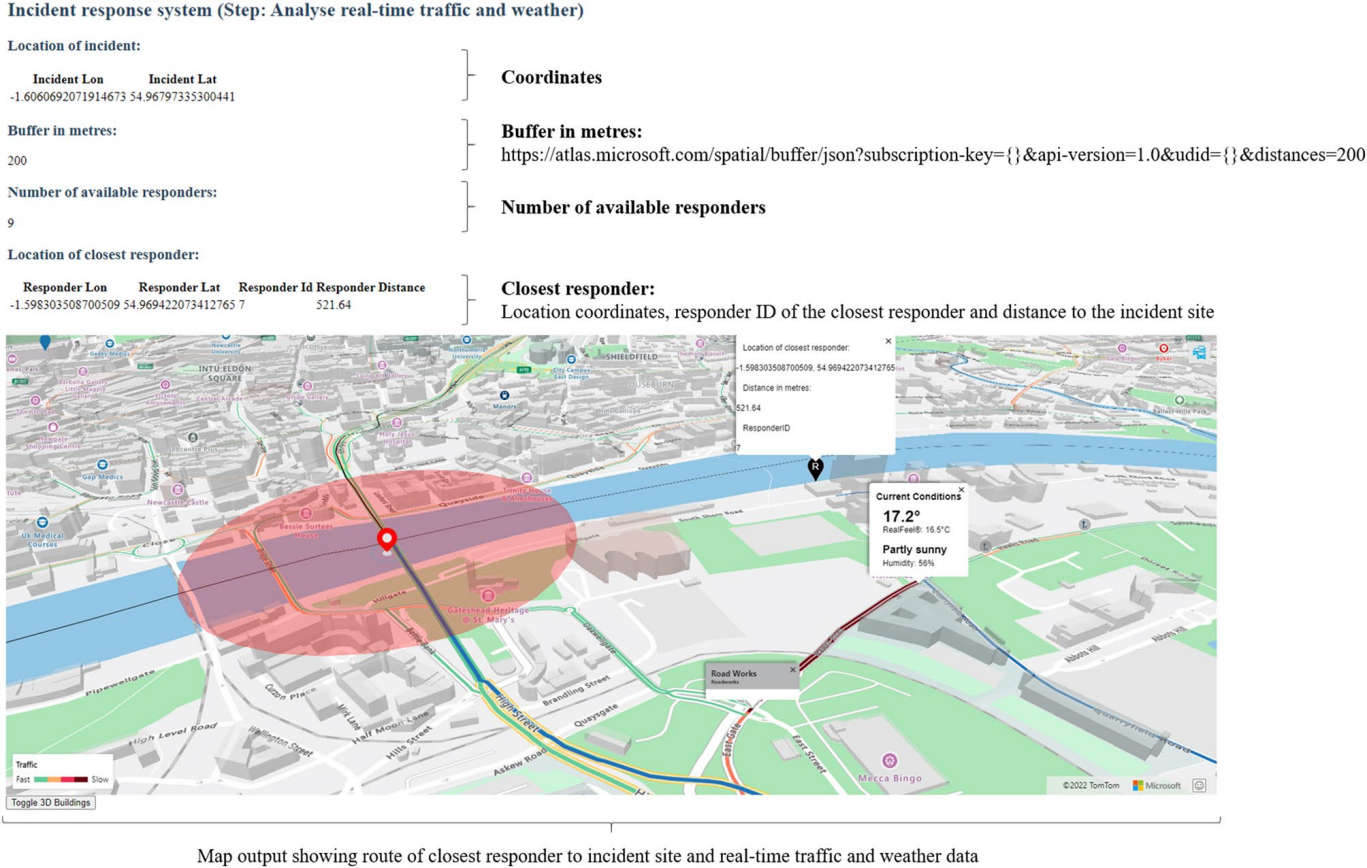
Incident process steps 4–6: Route navigation from closest responder to incident site using real-time traffic and weather data (own figure). The web application shows the indicators: Location of incident (longitude, latitude); buffer in metres; the number of available responders; and the location of the closest responder (responder longitude and latitude, responder ID, responder name and distance to the incident site). The map output visualises the location of the incident on the Tyne Bridge with a red marker and a red circular buffer area around the incident location. The black marker with the white letter "R" near the river indicates the closest responder to the incident site. The blue-marked road indicates the route in the network leading from the location of the responder to the incident site. The road network shows the traffic flow using the color ramp from green (fast) to red (slow). The pop-up windows show the current weather and road network conditions. KW created the map output using the Microsoft Azure Maps Web Software Development Kit and TomTom ( ^©^ 2022) base map data (a subscription key to use the data can be obtained after registering on the Azure Maps platform for Geospatial Mapping APIs under https://azure.microsoft.com/en-us/services/azure-maps/#azuremaps-overview). The code for the web application was developed in JavaScript, CSS and HTML in the Microsoft Visual Studio Code integrated development environment (version 1.70.2) and Node.js (version 16.13.2) on a Windows NT 64-bit operating system (×64-based processor)^40.^

Figure 5 visualises the output of the system prototype for the steps 4, 5, and 6 of the incident response process.

**Figure 6:**
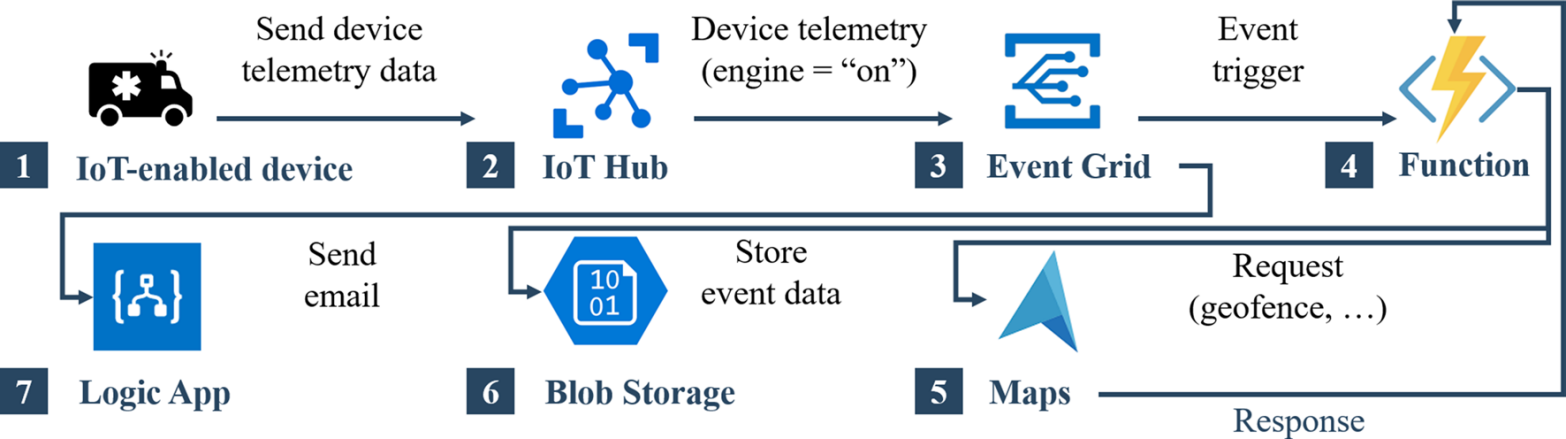
Incident process steps 7–8: Microsoft Azure workflow for geofencing analysis of responder vehicle to the incident site (own figure created using Microsoft PowerPoint and Azure icons^44^). To simulate and monitor an IoT-enabled device and inform stakeholders when the vehicle approaches the incident site, the following Microsoft Azure services are required: (1) IoT-enabled device; (2) IoT Hub; (3) Event Grid; (4) Function; (5) Azure Maps; (6) Blob Storage; and (7) Logic App.

For the steps (7) and (8), Figure 6 illustrates how the Azure services ^44^ described are implemented and interconnected to develop the use case and support stakeholders in incident response. The numbering in the figure refers to the following explanations.

1. *IoT-enabled device:* We simulate an ambulance vehicle equipped with an internet-enabled device to send telemetry data and further incident-relevant information. For the ambulance vehicle to send telemetry and further incident-relevant data to the IoT Hub, we must register the device with the Azure IoT Hub. Following the device registration, the ambulance vehicle can now send simulated telemetry data from the internet-enabled device to the IoT Hub.
2. *IoT Hub:* The IoT Hub acts as a central message hub for bidirectional communication between an IoT app and the devices it manages. If the engine is running (engine = “on”), the IoT Hub will publish telemetry data to the Event Grid. If the engine is not running (engine = “off”), the IoT Hub will not publish telemetry data.
3. *Event Grid*: The Event Grid trigger invokes a Function when the Event Grid sends an event (i.e. we have an event subscription to the telemetry data and can receive events sent by internet-enabled devices registered through the IoT Hub). We register an event subscription to the device telemetry data sent by the ambulance, which triggers a Function.
4. *Function:* When a specific event occurs, it triggers a function through the Event Grid. Here, an event characterises a device telemetry event, which occurs when our simulated device sends location coordinates of the responder vehicle. The event calls a Function as an endpoint, which receives the relevant data for the device registered in the IoT Hub. The Function stores the data received from the event (vehicle’s location coordinates, event time, and device ID) into a Blob Storage.
5. *Azure Maps:* The Function uses a Spatial Get Geofence API service to obtain information on whether the vehicle is within the pre-defined geofence area (here: incident site).
6. *Blob Storage:* We use the binary large object storage solution to store the telemetry data from the responder vehicle in .geojson format.
7. *Logic App:* We deploy a Logic App to create and run automated workflows. Here, we implement two Logic Apps to monitor when the responder vehicle enters the initial geofence (i.e. incident site) and exits the geofence. The Event Grid calls the corresponding Logic App endpoint that initiates the workflow to send a notification to other responders.

## Discussion

Multi-agencies need access to the same data and information about the current situation in a rapidly evolving incident situation. Due to the various domains, historical examples demonstrate challenges in interconnected multi-agency relationships, such as lacking relevant information and collaboration across agencies^45^. However, we also must recognise that a significant aspect of coordination is human. Thus, incidents that initially occur within the responsibility of one stakeholder domain can have long-term impacts on the broader city network^46^. Therefore, developing the prototype collaboratively with end users from multiple agencies is critical to limit the risks of failed collaboration that result from a system designed for only one end user.

This study presents the first design and prototype of an incident response system as part of an iterative systems engineering study. A network of spatially distributed sensors can help to assess the incident impact by monitoring the environment in real-time, comparing measurements to historical time-series data, and enabling disaster prevention through anomaly detection and pattern recognition. For such holistic, multi-agency impact analysis and better decision making, a smart city relies on data from multiple sources, which requires real-time fusion of various data streams^19,47^. For instance, during a storm event affecting various city domains, stakeholders can receive transport updates from CCTV cameras and sensors^48^, Twitter posts (about ongoing road incidents), rainfall data^49^, flood warnings^50^, and stakeholder calls. Although all data are in different formats, they all include incident-relevant information that needs to be aggregated in real-time to help responders choose their route when navigating to an incident. Thus, synthesising the different heterogeneous data streams and disseminating the information to relevant stakeholders can lead to more sustainable incident response and better situational awareness. In a connected and distributed multi-tenant environment, internet-based workflows to transmit continuous streams of telemetry data can fail within the system boundaries of individual stakeholders to entire cities. Following Azure’s design principles, this prototype must ensure reliability across different regions and zones and scalability across various subscriptions to reduce the impact of a single resource failure and mitigate data loss^51^. Further, we must monitor all registered devices; moreover, backend services must be able to automatically capture any issues in the incident management workflow to inform other involved agencies in case of device failure^52^.

The current prototype presents initial functionalities identified in the first phase of the literature review and stakeholder requirements through semi-structured interviews. The overarching goal of the development is to demonstrate and better understand how a single incident in one system can impact other systems. The initial prototype shows what interdependencies exist and what cascading impact incidents can have across the city and systems^53^. The developed use case helps to reflect on the challenges of data integration and the role of spatial data infrastructure in a multi-agency environment. The prototype developed can help to detect (near) real-time monitoring of the incident environment and work towards the user requirements shown in Table 2 in the different areas: (1) *Processes*: Stakeholders can receive automatic alerts from multi-agencies based on the nature of the incident and the status of the response. Further, stakeholders can send and receive the estimated time of arrival from multi-agencies at the incident site. Stakeholders also have a better joint overview, as they can use geofencing analysis to monitor when a responder vehicle entered or left the incident site. (2) *People*: Communication can be improved across multi-agencies. Stakeholders can receive status updates on changing conditions, e.g. a message can be sent to traffic managers when the incident site is cleared, and traffic can resume. (3) *Data*: Stakeholders have access to real-time traffic data provided by TomTom and real-time weather and hazard data provided by AccuWeather. *Technology*: The Microsoft Azure cloud computing technology enables the integration of heterogeneous data sources and provides various services that support an automated workflow for storing and analysing large, diverse datasets. (5) *Analytics*: Further development will include real-time analytics. Queries will be implemented to show analysis results on currently impacted areas and people at risk, and provide real-time people and vehicle counts for the areas.

This research demonstrates innovative spatial methods that utilise available cloud computing technologies to link heterogeneous, disparate data sources from various distributed systems, integrate real-time data, and provide various spatial analyses to diverse multiple end-users. Reflecting on the system design (shown in Fig. 1), the implemented prototype supports automated workflows, enables multi-agency collaboration, and helps to collaborate response to review earlier decisions using a feedback loop: (1) Incident handling: Stakeholders can receive geographical data about the incident and responder location data in .geosjon to capture an incident. (2) Data extraction and processing: Stakeholders can connect to internet-enabled devices and extract relevant telemetry and incident data. (3) Data analysis and visualisation: Using this data, stakeholders can perform different analysis, such as identifying available resources, determining the closest responder and visualising different locations on a map. (4) Data intelligence: Stakeholders can identify the closest responder from the incident site and calculate the route using real-time weather and traffic data. Further, other stakeholders can be notified about the estimated time of arrival. The system provides iterative updates until the response is performed and the incident resolved.

The system developed in this study is still a prototype designed and developed according to the ISO/IEC 25010 guidelines, which introduce different quality requirements and evaluation criteria^37^. Part of the process is demonstrating the technology and collaboratively developing an appropriate user interface with initial consultation and input from a group of relevant stakeholders. As a result, the prototype cannot be fully evaluated for criteria, such as usability at this stage. Early conversation with stakeholders showed that the visual representation of the map and the ETA were very useful across agencies. Further interface development requires refining the feedback process and collecting more in-depth user perspectives about different usability aspects based on the existing prototype.

## Conclusion

Incident management requires multi-agency collaboration, as impacts resulting from natural disasters such as floods or traffic accidents can cross administrative boundaries. In this study, we bring together a wide range of different stakeholders from different fields related to emergency and disaster response and ensure that different perspectives are integrated. While the focus of this study was on integrating these data sources for a specific problem, the underlying technology can also be used in other areas. A key criterion is that the technology is tailored to and co-developed with the end users in mind. This may result in changes to the visualisation, but the actual prototyping process and technology behind the data integration do not need to change as much. In this sense, this methodology can serve as an enabler and support in interdisciplinary and complex project environments. Further development will leverage the strategic value of hidden IoT data, such as real-time traffic data and people movement, and provide improved real-time contextual incident response for a smart, sustainable, and resilient city.

## Full methods

We first give a general description of the individual steps in the systems engineering approach^33^ and then describe how we apply the individual steps to our study:

1. *Defining the research problem:* Systems are becoming increasingly complex, dynamic, interconnected, and automated. The most important task in any system decision-making process is to understand the problem and define a clear problem statement before developing a solution. We look at the generic steps involved in responding to and managing incidents and define current challenges for multi-agency incident response based on literature review and stakeholder discussions.
2. *Identifying stakeholder needs:* Stakeholders are people who are interested in the solution and can influence the decision-making (e.g. the future software users). We hold discussions with the police, fire and rescue services, government departments and agencies, local authorities, the Environment Agency and other groups involved in operational incident response, data collection and management. The stakeholder analysis helps to better understand the problem at hand, and identify the needs, functions, objectives, and constraints of the different stakeholder groups (see Table 1).
3. *Analysing functional requirements:* Requirements describe technical capabilities requested in a system, while functions describe tasks, actions, or activities that must be performed to achieve the defined outcome. A requirements analysis determines specific characteristics of a system based on the previously identified stakeholder needs. Table 2 describes the functional user requirements for multi-agency incident response based on the discussions held with the stakeholders.
4. *Developing system design:* A preliminary system solution is designed and developed according to the predefined requirements, consisting of functional and technical elements. We design a conceptual workflow for a use case involving an incident at a critical transport link (Fig. 1) and explain the individual steps of the multi-agency response in Fig. 2.
5. *Feedback system design:* The preliminary system design is verified and validated using stakeholder feedback. We use this feedback to integrate future requirements into the system design during further development.
6. *Developing system prototype:* We develop a system prototype that meets the previously identified stakeholder requirements and present screenshots of the incident response application (Figs. 3, 4, 5) showing different steps.

The semi-structured interviews included questions on the following areas: *Processes*: Is there currently a framework available for incident response? What are the steps in incident response? How can the process design be improved? *People*: What other stakeholders are involved in the incident process? How do you work together? How do you communicate with other stakeholders? Do you have access to the same incident-related data? *Data*: What data do you require for incident response? How do you receive and access the data? How do you receive incident-related updates? How do you share updates with others during the incident response? Do you have access to real-time data? *Technology*: What technologies and systems do you use? Does the technology support the processing of real-time information? *Analytics*: What analytical capabilities does your current system provide? What analytical capabilities can support future incident response? What other data is required for that?

A Microsoft Azure account is required to create and configure the Azure Maps, IoT Hub, Event Grid, Function, Blob storage and Logic App^40^. To simulate location data for an internet-enabled device along a network route, we deploy an Azure IoT C# application^41^. The incident response system prototype is developed using Azure Maps, HTML5, JavaScript, and CSS. We use the Azure Maps REST API services: Data Upload, Spatial (Buffer, Closest Point, Geofence), Route Service, AccuWeather service, TomTom traffic service^42^. We simulate incident data (longitude and latitude coordinates and incident type) and data of first responders (longitude and latitude coordinates, responder ID and responder name) data in .geojson format.

## Data Availability

The datasets generated during and/or analysed during the current study are available from the corresponding author on reasonable request.
